# Impact of the first wave of COVID-19 on Crohn’s disease after the end of “zero-COVID” policy in China

**DOI:** 10.3389/fpubh.2023.1186275

**Published:** 2023-07-13

**Authors:** Wen Hu, Xiao Li, Zelin Yan, Qiuzhi Wang, Jiakai Luo, Qiao Yu, Shuyan Li, Shiyuan Lu, Atiyeh Roozbahani, Ehsan Ghoushi, Yan Chen, Jun Li

**Affiliations:** ^1^State Key Laboratory for Diagnosis and Treatment of Infectious Diseases, National Clinical Research Center for Infectious Diseases, National Medical Center for Infectious Diseases, Collaborative Innovation Center for Diagnosis and Treatment of Infectious Diseases, The First Affiliated Hospital, Zhejiang University School of Medicine, Hangzhou, China; ^2^The China Crohn’s & Colitis Foundation, Hangzhou, China; ^3^Department of Pathology, The First Affiliated Hospital, Zhejiang University School of Medicine, Hangzhou, China; ^4^Center for Inflammatory Bowel Disease, Department of Gastroenterology, The Second Affiliated Hospital, Zhejiang University School of Medicine, Hangzhou, China; ^5^Department of Nursing, The Second Affiliated Hospital, Zhejiang University School of Medicine, Hangzhou, China; ^6^Zhejiang University School of Medicine, Hangzhou, China

**Keywords:** COVID-19, Crohn’s disease, cohort study, China, “zero-COVID” policy

## Abstract

**Background:**

The incidence and severity of coronavirus disease 2019 (COVID-19) among Crohn’s disease (CD) patients are unknown in China. This study aimed to clarify the clinical courses and outcomes of CD patients in the first COVID-19 wave after the end of “zero-COVID” policy in China.

**Methods:**

Clinical characteristics, including vaccination doses and medications of 880 CD patients from a prospective cohort were collected for analysis.

**Results:**

Of the enrolled patients (*n* = 880) who underwent nucleic acid or antigen testing for COVID-19 from Dec 7, 2022, to Jan 7, 2023, 779 (88.5%) were infected with COVID-19. Among the infected patients, 755 (96.9%) were mild, 14 (1.8%) were moderate, one patient with leukemia died of cerebral hemorrhage (mortality, 0.1%) and only 9 (1.2%) were asymptomatic. Fever, cough, headache and appetite loss were the most frequently observed symptoms in general, respiratory, neurological and gastrointestinal manifestations, respectively. The age and disease duration were significantly higher (40/32, 5.6/3.6, all *p* < 0.05) in moderate patients than those in mild patients. All other clinical characteristics, including CD activity and medication exposure, showed no significant differences between the above two groups. Furthermore, no significant difference in vaccination or comorbidities was observed between the two groups.

**Conclusion:**

Most CD patients contracted the Omicron infection and experienced mild disease courses in the first COVID-19 wave attack after China ended the “zero-COVID” policy irrespective of vaccination dose or comorbidities.

## 1. Introduction

Crohn’s disease (CD), one main type of inflammatory bowel disease (IBD), is a chronic inflammatory condition of the gastrointestinal tract with a relapsing–remitting and progressively disabling pattern (1). The management of CD during the coronavirus disease 2019 (COVID-19) pandemic has been a research priority for the IBD community worldwide over the last 3 years (2). Patients with CD, especially in the presence of immunosuppressive medications, are supposed to be at high risk of serious viral and bacterial infections (3). Evidence from studies in the phases of earlier variants (alpha, beta, gamma, and delta) revealed no differences in COVID-19 hospitalization or mortality between patients with IBD or without IBD (4), while advanced age and the presence of comorbid conditions were found to be key risk factors for severe infection (5). Very few data concerning the impact of the new Omicron strain with high transmissibility on CD patients have been reported (6–8). COVID-19 vaccines are believed to play a protective role against severe acute respiratory syndrome coronavirus 2 (SARS-CoV-2); however, concerns about vaccination efficacy are one reason for hesitancy (9). Along with the adjustment of the “zero-COVID” strategy in China on 7 December 2022, the first nationwide Omicron-based outbreak started shortly after the relaxation of nonpharmaceutical public health intervention measures (including social distancing, mass testing, quarantine and travel restrictions), and passed the peak rapidly within 1 month (Dec 8, 2022 to Jan 7, 2023) with more than 50,000 deaths.^1^ The impact of this on CD patients who were naïve to COVID with different vaccination backgrounds should be clarified to promote our understanding of COVID-19 and CD management. This study aimed to clarify the clinical courses and outcomes of CD patients in the first COVID-19 wave after the end of “zero-COVID” policy in China.

## 2. Patients and methods

### 2.1. Study design

**Figure 1:**
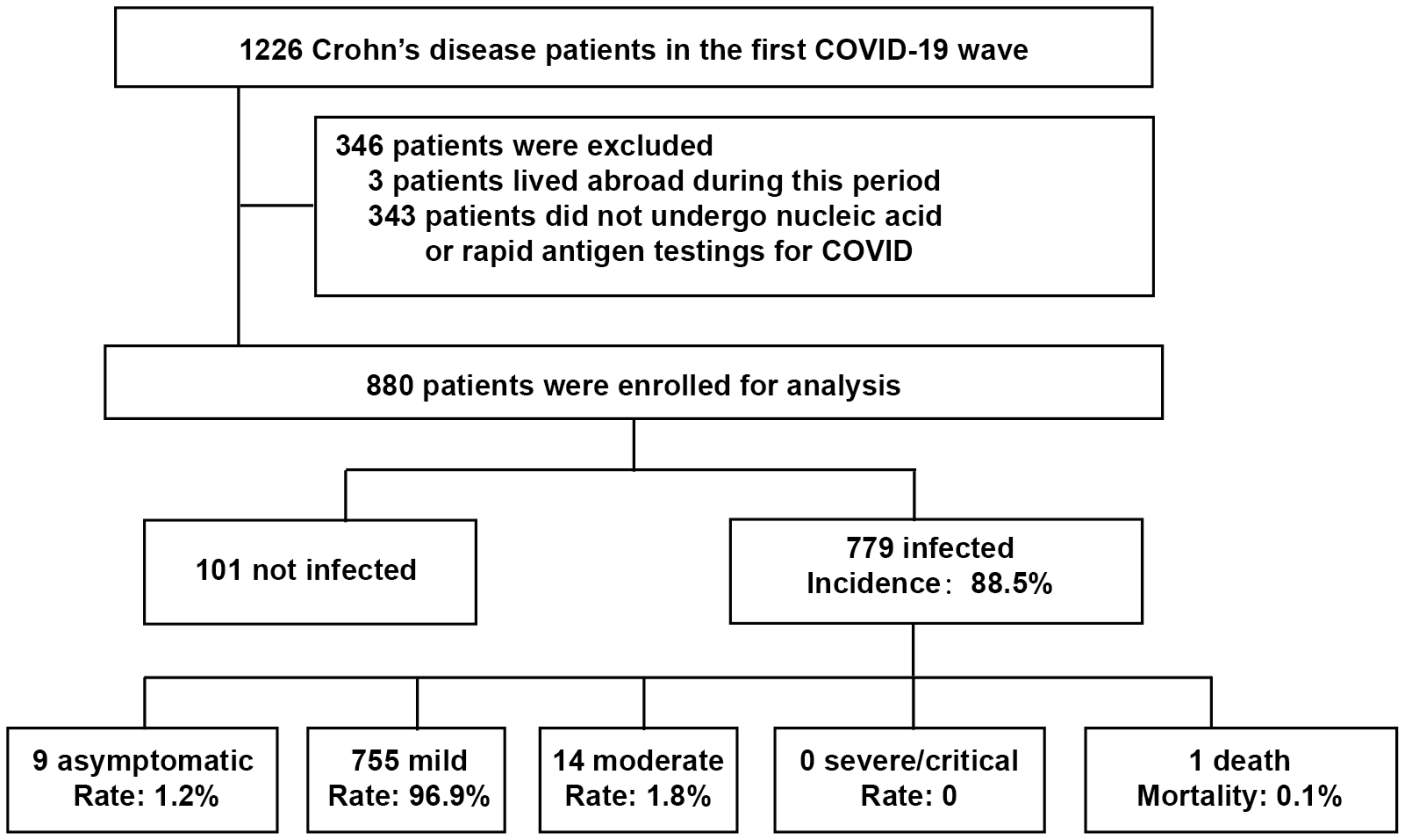
Study population flowchart. COVID-19, coronavirus disease 2019.

CD patients from our prospective open cohort (established from July 1st, 2019) who had nucleic acid or rapid antigen tests during the first wave (Dec 8, 2022, to Jan 7, 2023) were enrolled in this study (Figure 1). Clinical data, including comorbidities, medications and vaccinations, were collected from the cohort database and follow-up information. The incidence and severity of COVID-19 among CD patients were analyzed. This study was approved by the Institutional Review Board of the Ethics Committee of the Second Affiliated Hospital, School of Medicine, Zhejiang University in China (approved No. 2023-0134). In all cases, informed written consent was obtained from participants or their legal surrogates before enrollment. The study followed the STROBE reporting guideline.

### 2.2. Patients

CD was diagnosed based on a combination of clinical, laboratory, endoscopic, cross-sectional imaging, and histological assessments. The exclusion criteria were as follows: (1) patients who were living abroad during the first wave; or (2) patients who did not undergo any nucleic acid or rapid antigen tests for COVID-19. All patients in the cohort were followed up in a short time to reduce recall bias and underreported bias.

### 2.3. Criteria for COVID-19 diagnosis and severity

COVID-19 diagnosis was based on viral tests (by nucleic acid or rapid antigen tests, irrespective of symptoms) and disease severity was classified according to the ninth edition of the COVID-19 diagnosis and treatment protocol (10). Symptomatic COVID-19 infections were defined as those that had a positive nucleic acid or rapid antigen test result with at least one of 22 symptoms (11). Asymptomatic infections were defined as a positive nucleic acid or rapid antigen test without any symptoms at the time of testing. Mild infection was defined as slight clinical symptoms without pneumonia on chest imaging. Moderate infection was defined as clinical symptoms plus pneumonia on chest imaging without evidence of hypoxia. Severe infection was diagnosed according to dyspnea (respiratory rate ≥ 30 times/min), resting finger oxygen saturation ≤ 93%, or artery PaO2/FiO2 ≤ 300 mm Hg (1 mm Hg = 0.133 kPa). Critical infection was defined as respiratory failure with shock and multiorgan failure requiring mechanical ventilation and intensive care unit admission.

### 2.4. Data collection and procedure

Demographic (including sex and age) and diagnostic profiles (diagnosis, disease duration and chronic illness history) were extracted from the cohort database. The following information was collected during follow-ups: COVID-19 diagnosis status, COVID-19 symptoms, body mass index (BMI), COVID-19 vaccination doses, disease activity prior to COVID-19 infection [as defined by the Harvey-Bradshaw Index, HBI (12)], medication exposure at time of COVID-19 diagnosis and whether medications were discontinued, chest imaging and COVID-19 treatments. Patients with COVID-19 diagnosed less than 7 days prior were followed up again to confirm any progression on Jan 20th, 2023.

### 2.5. Statistical analysis

For continuous variables, the means (standard deviations, SDs) and medians (interquartile ranges, IQRs) were used for normally and nonnormally distributed data, followed by unpaired t tests and Mann–Whitney *U* tests when appropriate. Categorical variables were expressed as numbers (%) and compared using Fisher’s exact test. *p* < 0.05 was considered statistically significant and SPSS (V.26.0) was used for all analyses.

## 3. Results

A total of 1,226 CD patients were extracted from the cohort database and 880 patients were enrolled for the final analysis. Of the enrolled patients, 779 (88.5%) were diagnosed with COVID-19 infection, of whom, 9 (1.2%) were asymptomatic, 755 (96.9%) were mild, 14 (1.8%) were moderate, and one patient with leukemia died of cerebral hemorrhage after COVID-19 infection (Figure 1 and Table 1).

**Table 1:** Clinical characteristics of Crohn’s disease patients enrolled for COVID-19 analysis.

Characteristic	Total (*n* = 880)	Infection (*n* = 779)	Non-infection (*n* = 101)	*p* value
Sex, *n* (%)				0.729
Male	620 (70.5)	547 (70.2)	73 (72.3)	
Female	260 (29.5)	232 (29.8)	28 (27.7)	
Age in years	32 [26–41]	32 [26–41]	32 [25–41]	0.698
14–19	40 (4.5)	35 (4.5)	5 (5.0)	0.799
20–29	305 (34.7)	267 (34.3)	38 (37.6)	0.507
30–39	291 (33.1)	262 (33.6)	29 (28.7)	0.369
40–49	131 (14.9)	115 (14.8)	16 (15.8)	0.767
50–59	76 (8.6)	67 (8.6)	9 (8.9)	0.852
60–69	28 (3.2)	24 (3.1)	4 (4.0)	0.552
> = 70	9 (1.0)	9 (1.2)	0	>0.999
CD duration (years)	3.6 [1.6–6.6]	3.6 [1.6–6.5]	3.1 [1.5–7.5]	0.803
<1	119 (13.5)	105 (13.5)	14 (13.9)	0.878
1–5	432 (49.1)	384 (49.3)	48 (47.5)	0.752
5–10	225 (25.6)	199 (25.5)	26 (25.7)	>0.999
>10	104 (11.8)	91 (11.7)	13 (12.9)	0.743
BMI, kg/m^2^	21.0 [19.0–23.4]	21.0 [19.0–23.5]	21.1 [19.1–22.4]	0.275
≤18.4	160 (18.2)	141 (18.1)	19 (18.8)	0.891
18.5–23.9	547 (62.2)	477 (61.2)	70 (69.3)	0.127
24–27.9	146 (16.6)	136 (17.5)	10 (9.9)	0.064
≥28	27 (3.1)	25 (3.2)	2 (2.0)	0.759
Vaccination, *n* (%)				
No vaccination	194 (22.0)	165 (21.2)	29 (28.7)	0.097
One dose	43 (4.9)	39 (5.0)	4 (4.0)	0.809
Two doses	230 (26.1)	210 (27.0)	20 (19.8)	0.148
Three doses	394 (44.8)	347 (44.5)	47 (46.5)	0.75
Four doses	19 (2.2)	18 (2.3)	1 (1.0)	0.714
Current smoker, *n* (%)	60 (6.8)	50 (6.4)	10 (9.9)	0.206
Current pregnancy, *n* (%)	5 (0.6)	3 (0.4)	2 (2.0)	0.104
Comorbid conditions, *n* (%)				
Any comorbidity	135 (15.3)	119 (15.3)	16 (15.8)	0.999
Cancer	8 (0.9)	8 (1.0)	0	0.607
Hypertension	27 (3.1)	25 (3.2)	2 (2.0)	0.759
Diabetes	4 (0.5)	3 (0.4)	1 (1.0)	0.386
Cardiovascular disease	9 (1.0)	7 (0.9)	2 (2.0)	0.277
Lung disease	14 (1.6)	11 (1.4)	3 (3.0)	0.211
Chronic renal disease	3 (0.3)	3 (0.4)	0	>0.999
Chronic HBV infection	49 (5.6)	45 (5.8)	4 (4.0)	0.644
History of stroke	4 (0.5)	3 (0.4)	1 (1.0)	0.386

Categorical variables are expressed as *n* (%); continuous variables are expressed as the medians [Q1-Q3]. Comparisons were applied between the infection and non-infection group (Mann–Whitney *U* test or Fisher’s exact test). COVID-19: coronavirus disease 2019; CD: Crohn’s disease; HBV: hepatitis B virus.

The clinical characteristics of all enrolled patients are summarized in Table 1. Among 880 CD patients, 620 (70.5%) were male. The age and disease duration distributions were left-skewed, with a median age of 32 (26–41) years and a CD duration of 3.6 (1.6–6.6) years. The median BMI was 21.0 (19.0–23.4). A total of 43 (4.9%), 230 (26.1%), 394 (44.8%) and 19 (2.2%) patients had been vaccinated with one dose, two doses, three doses and four doses, respectively, while 194 (22.0%) patients were not vaccinated. Sixty patients (6.8%) were current smokers and 5 (0.6%) were pregnant during the study period. Fifteen percent of patients had at least one comorbidity in addition to CD, the most common being chronic hepatitis B virus infection (5.6%) and hypertension (3.1%). Comparative analysis between the infection and non-infection groups showed no difference in sex, age, disease duration, vaccination doses or comorbidities.

**Figure 2:**
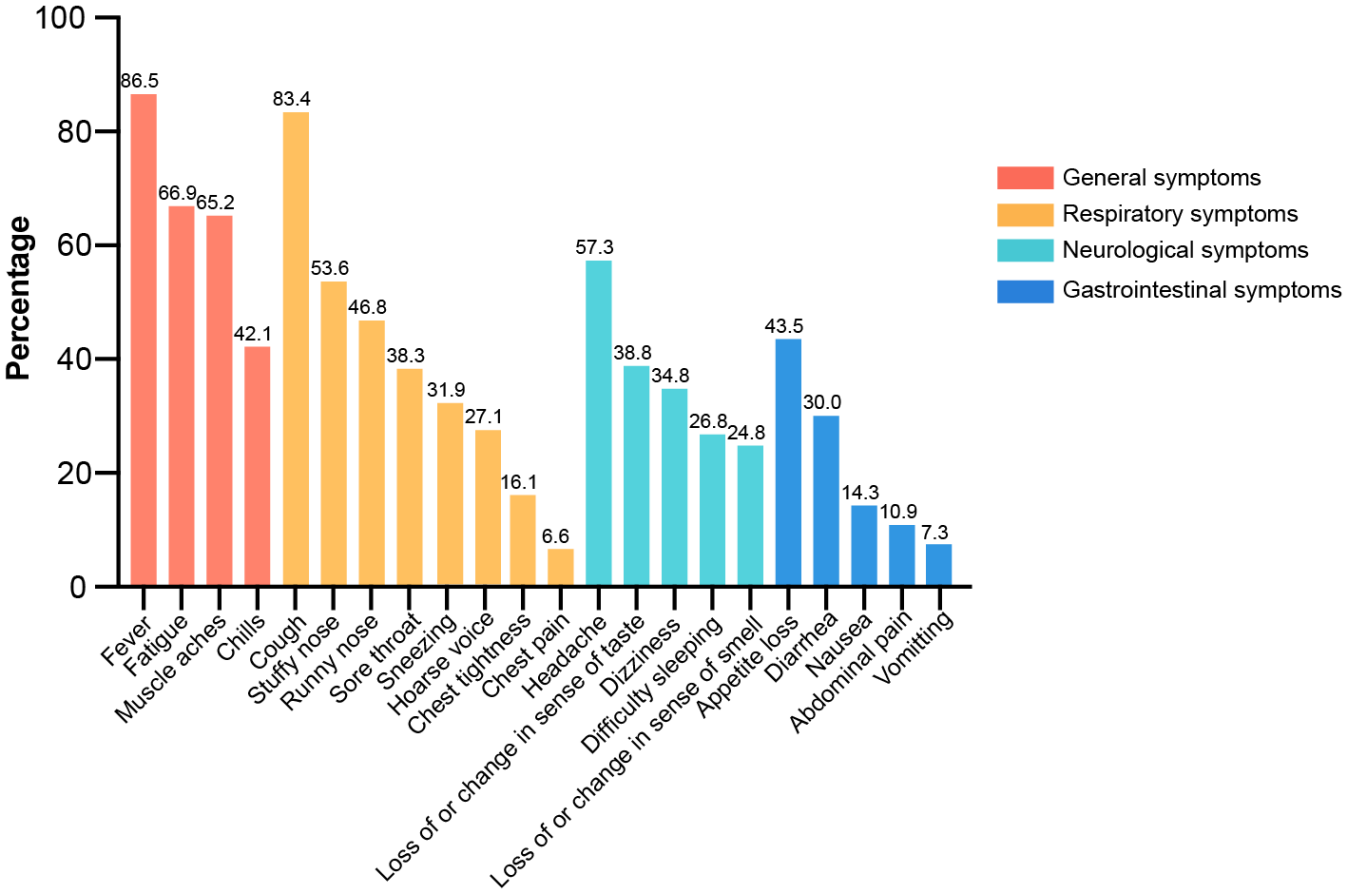
Categorization and ranking of symptom profiles.

All 22 clinical symptoms were summarized and ranked by their prevalence among symptomatic patients under categories of general, respiratory, neurological, and gastrointestinal (GI) manifestations in Figure 2. General symptoms were mostly reported, under which fever (86.5%) was the leading complaint followed by fatigue (66.9%), muscle aches (65.2%) and chills (42.1%). Manifestations of the respiratory system had the widest spectrum of symptoms, such as cough (83.4%), stuffy nose (53.6%), runny nose (46.8%), sore throat (38.3%), sneezing (31.9%), hoarse voice (27.1%), chest tightness (16.1%) and chest pain (6.6%). Neurological manifestations were the third most common symptoms, including headache (57.3%), loss of or change in sense of taste (38.8%), dizziness (34.8%), difficulty sleeping (26.8%) and loss of or change in sense of smell (24.8%). GI complaints were ranked as the fourth most common discomfort and included appetite loss (43.5%), diarrhea (30.0%), nausea (14.3%), abdominal pain (10.9%) and vomiting (7.3%). Collectively, fever, cough, headache, and appetite loss were the most observed symptoms in terms of general, respiratory, neurological and GI manifestations, respectively.

The characteristics of CD patients with symptomatic COVID-19 infections are summarized in Table 2 (*n* = 769, one death was excluded) and grouped by disease severity. Seventy percent patients were male, with a median age of 32 (26–40) years. The median CD duration was 3.6 (1.6–6.5) years, and the median BMI was 21.0 (19.0–23.5). Only 42 (5.5%) patients were in an active state (HBI > 3) prior to COVID-19 infection. Apart from the common gastrointestinal symptoms including diarrhea and abdominal pain, some patients presented other fluctuations in CD-related symptoms, including abdominal mass (0.5%), constipation (0.3%), hematochezia (0.8%), bloating (0.5%), perianal symptoms (0.7%) and extraintestinal manifestations (1.3%). Medication exposure was summarized in commonly used categories. Tumor necrosis factor (TNF) antagonists (55.4%) was mostly used, followed by ustekinumab (20.5%) and vedolizumab (2.3%) in the biologics class. Three kinds of immunomodulators were used, including thiopurine (17.2%), methotrexate (2.7%) and thalidomide (1.8%). Only 7 (0.9%) and 2 (0.3%) patients were using corticosteroids and tofacitinib, respectively, before infection. Thirty-nine (5.1%) patients were taking sulfasalazine/mesalamine. Concerning medications for COVID-19, acetaminophen (50.8%) was the most frequently used drug, followed by Chinese patent medicine (23.9%) and anti-cough drugs (22.4%). Between-group comparisons showed that the moderate group was older (40 vs. 32, *p* < 0.05) and had a longer CD disease duration (5.6 vs. 3.6, *p* < 0.05) than the mild group. All other clinical characteristics, including CD activity and medication exposure, showed no significant differences between the above two groups.

**Table 2:** Clinical characteristics of Crohn’s disease patients with symptomatic COVID-19 infection.

Characteristic	Total (*n* = 769)^a^	Mild (*n* = 755)	Moderate (*n* = 14)	*p* value
Male, *n* (%)	539 (70.1)	530 (70.2)	9 (64.3)	0.769
Age (years)	32 [26–40]	32 [26–40]	40 [31–43]	**0.041**
CD duration (years)	3.6 [1.6–6.5]	3.6 [1.6–6.5]	5.6 [3.4–13.1]	**0.042**
BMI, kg/m^2^	21.0 [19.0–23.5]	21.0 [19.0–23.5]	22.4 [19.1–25.8]	0.244
CD activity (HBI) prior to infection, *n* (%)		0.476
0	505 (65.7)	494 (65.4)	11 (78.6)	
1–3	222 (28.9)	220 (29.1)	2 (14.3)	
>3	42 (5.5)	41 (5.4)	1 (7.1)	
Fluctuation of activity, *n* (%)				
Any new symptoms	330 (42.9)	322 (42.6)	8 (57.1)	0.416
Abdominal pain	84 (10.9)	81 (10.7)	3 (21.4)	0.190
Diarrhea	231 (30.0)	226 (29.9)	5 (35.7)	0.769
Nausea	110 (14.3)	110 (14.6)	0	0.240
Vomiting	56 (7.3)	56 (7.4)	0	0.615
Abdominal mass	4 (0.5)	4 (0.5)	0	>0.999
Constipation	2 (0.3)	2 (0.3)	0	>0.999
Hematochezia	6 (0.8)	6 (0.8)	0	>0.999
Distension/bloating	4 (0.5)	4 (0.5)	0	>0.999
Perianal symptoms	5 (0.7)	5 (0.7)	0	>0.999
Extraintestinal manifestations	10 (1.3)	9 (1.2)	1 (7.1)	0.169
Medications for CD, *n* (%)				
TNF antagonists	426 (55.4)	418 (55.4)	8 (57.1)	>0.999
Ustekinumab	158 (20.5)	155 (20.5)	3 (21.4)	>0.999
Vedolizumab	18 (2.3)	18 (2.4)	0	>0.999
Thiopurine	132 (17.2)	129 (17.1)	3 (21.4)	0.718
Methotrexate	21 (2.7)	20 (2.6)	1 (7.1)	0.324
Thalidomide	14 (1.8)	14 (1.9)	0	>0.999
Corticosteroids	7 (0.9)	7 (0.9)	0	>0.999
Tofacitinib	2 (0.3)	2 (0.3)	0	>0.999
Sulfasalazine/mesalamine	39 (5.1)	39 (5.2)	0	>0.999
Medications for COVID-19, *n* (%)				
Acetaminophen	391 (50.8)	384 (50.9)	7 (50.0)	>0.999
NSAIDs	151 (19.6)	149 (19.7)	2 (14.3)	>0.999
Chinese patent medicine	184 (23.9)	178 (23.6)	6 (42.9)	0.113
Traditional Chinese medicine	23 (3.0)	22 (2.9)	1 (7.1)	0.349
Anti-cough drugs	172 (22.4)	164 (21.7)	8 (57.1)	**0.005**
Anti-diarrheal drugs	14 (1.8)	14 (1.9)	0	>0.999
Anti-viral drugs	5 (0.7)	4 (0.5)	1 (7.1)	0.088

Categorical variables are expressed as *n* (%); continuous variables are expressed as the medians [Q1-Q3]. Comparisons were applied between the mild and moderate group (Mann–Whitney *U* test or Fisher’s exact test).

a One patient who died of cerebral hemorrhage after COVID-19 infection was removed from the analysis. COVID-19, coronavirus disease 2019; CD, Crohn’s disease; HBI, the Harvey-Bradshaw Index; TNF, Tumor necrosis factor; NSAIDs, Nonsteroidal anti-inflammatory drugs.

Significant results are presented in bold.

The vaccination doses and comorbidities of symptomatic patients are summarized in Table 3. Five (35.7%) and 7 (50.0%) patients in the moderate group had been vaccinated with two doses and three doses, while 38 (5.0%), 203 (26.9%), 335 (44.4%) and 18 (2.4%) patients in the mild group had been vaccinated with one dose, two doses, three doses and four doses, respectively. The two leading comorbidities were chronic hepatitis B virus infection (5.4% in mild and 7.1% in moderate group) and hypertension (1.3% in mild and 7.1% in moderate group). There was no significant difference in either vaccination doses or comorbidities between patients in the mild and moderate groups.

**Table 3:** Vaccination and comorbidities of Crohn’s disease patients with symptomatic COVID-19 infection.

Characteristic	Total (*n* = 769)^a^	Mild (*n* = 755)	Moderate (*n* = 14)	*p* value
Vaccination, *n* (%)				
No vaccination	162 (21.1)	161 (21.3)	1 (7.1)	0.322
One dose	39 (5.1)	38 (5.0)	1 (7.1)	0.521
Two doses	208 (27.0)	203 (26.9)	5 (35.7)	0.543
Three doses	342 (44.5)	335 (44.4)	7 (50.0)	0.788
Four doses	18 (2.3)	18 (2.4)	0	>0.999
Current smoker, *n* (%)	48 (6.2)	48 (6.4)	0	>0.999
Comorbid conditions, *n* (%)				
Cancer	7 (0.9)	7 (0.9)	0	>0.999
Hypertension	24 (3.1)	23 (3.0)	1 (7.1)	0.361
Diabetes	2 (0.3)	2 (0.3)	0	>0.999
Cardiovascular disease	6 (0.8)	6 (0.8)	0	>0.999
Lung disease	11 (1.4)	10 (1.3)	1 (7.1)	0.184
Chronic renal disease	3 (0.4)	3 (0.4)	0	>0.999
Chronic HBV infection	42 (5.5)	41 (5.4)	1 (7.1)	0.548

Categorical variables are expressed as *n* (%); continuous variables are expressed as the medians [Q1-Q3]. Comparisons were applied between the mild and moderate group (Mann–Whitney *U* test or Fisher’s exact test).

a One patient who died of cerebral hemorrhage after COVID-19 infection was removed from the analysis. COVID-19, coronavirus disease 2019; HBV, hepatitis B virus.

## 4. Discussion

We used cohort-based clinical data and follow-up COVID-19 information to clarify the impact of Omicron on the infection-naïve CD population during the China’s first wave after the end of “zero-COVID” policy. The results showed that most CD patients experienced symptomatic infections and mild clinical courses.

The first nationwide COVID-19 wave in China started shortly after the implementation of measures, peaked in late December, then declined continuously and ended in late January^2^. From Dec 8th, 2022, to Jan 7th, 2023, the first wave of COVID-19 in China claimed more than 50,000 lives (see footnote 1). Although the dominant strains that drove the wave were Omicron BF.7 and BA.5.2, which were regarded as highly transmissible but low-virulence subvariants, COVID-19 still triggered great anxiety and stress among CD patients who had never been exposed to COVID. During the observation time, the incidence rate of COVID-19 among CD patients was 88.5%, which was approximately equal to that in the general population reported in Henan province in early January (urban 89.1%, rural 88.9%) (13). Among the infected patients, the majority of them experienced mild (96.9%) or moderate (1.8%) courses. Lu et al. recently reported that 96.97% of the general population infected with COVID-19 experienced mild or moderate symptoms during the same time of our study (14). This similar outcome may be associated with the younger age structure and use of biologics and immunosuppressants in our CD population (15). The lack of biosamples for further genetic analysis is our limitation, as CD patients were encouraged to follow home treatment during the pandemic. No severe/critical cases were observed in our study, and the only death case was caused by complications of leukemia, exacerbated by COVID-19. Given that hematological malignancies have a high mortality rate (29.3–40%) during COVID-19 (16, 17), these groups of patients should be a priority for protection in future outbreaks.

Age and comorbidities are the most important prognostic factors for more severe COVID-19 among IBD patients according to previous studies of earlier variants (18, 19). In the analysis of IBD medications, systemic corticosteroids, the combination of TNF antagonists with azathioprine and active IBD were associated with poor outcomes of COVID-19 (20). Older age and longer CD disease duration were found to be associated with moderate COVID-19, indicating that age and accumulated damage may influence the viral-induced immune response. All other clinical characteristics, including CD activity, medication exposure and comorbidities, showed no significant differences between the above two groups. Given the low rates of moderate/severe cases in our study, our findings need validation in large external cohorts.

The symptom profile reflects the potential of COVID-19 to damage multiple systems through immune responses (21), and changes with the evolution of variants (11). Consistent with the findings that influenza-like symptoms were more frequently reported in Omicron (11), this study demonstrated the highest prevalence of general symptoms among symptomatic patients. Fluctuation of CD-related symptoms was another concern for most patients and physicians during the infection. Over 40% of patients in our study reported fluctuations in CD-related symptoms, including common GI symptoms and CD-specific manifestations. In contrast to the data from Surveillance Epidemiology of Coronavirus Under Research Exclusion (22), patients in our cohort presented a higher rate of common GI symptoms (diarrhea 20.9% vs. 30.0%, abdominal pain 8.9% vs.10.9%); this needs further validation in external cohorts. It is necessary to prolong the observation time for activity patterns and outcomes among these patients.

Although vaccination against SARS-CoV-2 has been recommended to IBD patients since the beginning of the pandemic (23), the rate of uptake among our CD patients was approximately 75% which was lower than that in the general population, reflecting the phenomenon of vaccine hesitancy in this immunosuppressed population (24). Vaccine effectiveness against viral acquisition and severe outcomes was assessed in recent population-based studies during Omicron outbreaks, suggesting that a booster dose of COVID-19 vaccine is needed for older patients and high-risk populations against severe or fatal outcomes (25). Bivalent booster vaccines are now encouraged among IBD patients taking TNF antagonists and tofacitinib based on emerging evidence regarding the effectiveness of COVID-19 vaccines (8, 26). No significant difference in vaccination doses was observed between the infection and non-infection group or between the mild and moderate groups in this study despite the same infection-naïve background. It is worth noting that the vaccine effectiveness in IBD patients is influenced by many factors, such as vaccine type, doses, and waning antibodies with time. This study was limited by its retrospective design and inadequate sample size; therefore, future prospective studies with large cohorts are needed to evaluate the effectiveness and adjust the vaccination protocol for this special population.

In summary, our study reported the impact of COVID-19 on CD patients from a prospective cohort in the first countrywide wave after the end of “zero-COVID” policy in China. Most CD patients contracted the Omicron infection and experienced mild disease courses irrespective of vaccination dose or comorbidities. Our study presents clinicians with first-hand data on COVID-19 in CD patients during the first wave attack and may help ease the health anxiety of patients in the next wave of the pandemic.

## Data availability statement

The raw data supporting the conclusions of this article will be made available by the authors, without undue reservation.

## Ethics statement

The studies involving human participants were reviewed and approved by the Institutional Review Board of the Ethics Committee of the Second Affiliated Hospital, School of Medicine, Zhejiang University in China (approved No. 2023-0134). Written informed consent to participate in this study was provided by the participants’ legal guardian/next of kin.

## Author contributions

JL and YC: conceptualization, writing—review and editing, supervision, and funding acquisition. WH, XL, and ZY: methodology, software, visualization, and project administration. ZY: validation. WH and XL: formal analysis and data curation. XL, ZY, QW, JkL, QY, SuL, SiL, AR, and EG: investigation. YC and SuL: resources. WH, XL, ZY, QW, JkL, QY, and SiL: writing—original draft preparation. All authors contributed to the article and approved the submitted version.

## Funding

This study was supported by the National Natural Science Foundation of China (grant number 81830073), and the Qingfeng Scientific Research Fund of the China Crohn’s & Colitis Foundation (CCCF) (grant number CCCF-QF-2022B62-8).

## Conflict of interest

ZY was employed by The China Crohn’s & Colitis Foundation.

The remaining authors declare that the research was conducted in the absence of any commercial or financial relationships that could be construed as a potential conflict of interest.

## Publisher’s note

All claims expressed in this article are solely those of the authors and do not necessarily represent those of their affiliated organizations, or those of the publisher, the editors and the reviewers. Any product that may be evaluated in this article, or claim that may be made by its manufacturer, is not guaranteed or endorsed by the publisher.

## Footnotes

^1^ https://www.chinacdc.cn

^2^ https://weekly.chinacdc.cn/
